# Cadmium isotope fractionation in the soil – cacao systems of Ecuador: a pilot field study†

**DOI:** 10.1039/c9ra05516a

**Published:** 2019-10-23

**Authors:** Fiorella Barraza, Rebekah E. T. Moore, Mark Rehkämper, Eva Schreck, Grégoire Lefeuvre, Katharina Kreissig, Barry J. Coles, Laurence Maurice

**Affiliations:** Géosciences Environnement Toulouse (GET), Observatoire Midi-Pyrénées, CNRS, IRD, Université de Toulouse 14 avenue E. Belin F-31400 Toulouse France fiorella.barraza@hotmail.com; Department of Earth Science & Engineering, Imperial College London London SW7 2AZ UK markrehk@imperial.ac.uk r.moore13@imperial.ac.uk; Universidad Andina Simón Bolívar Área de Salud, P.O. Box 17-12-569 Quito Ecuador

## Abstract

The often high Cd concentrations of cacao beans are a serious concern for producers in Latin America due to the implementation of stricter Cd limits for cocoa products by the European Union in 2019. This is the first investigation to employ coupled Cd isotope and concentration measurements to study soil – cacao systems. Analyses were carried out for 29 samples of soils, soil amendments and cacao tree organs from organic farms in Ecuador that harvest three distinct cacao cultivars. The majority of soils from 0–80 cm depth have very similar δ^114/110^Cd of about −0.1‰ to 0‰. Two 0–5 cm topsoils, however, have high Cd concentrations coupled with heavy Cd isotope compositions of δ^114/110^Cd ≈ 0.2%, possibly indicating Cd additions from the tree litter used as organic fertilizer. Whilst cacao leaves, pods and beans are ubiquitously enriched in Cd relative to soils there are distinct Cd isotope signatures. The leaves and pods are isotopically heavier than the soils, with similar Δ^114/110^Cd_leaf–soil_ values of 0.22 ± 0.07‰ to 0.41 ± 0.09‰. In contrast, the data reveal differences in Δ^114/110^Cd_bean–leaf_ that may be linked to distinct cacao cultivars. In detail, Δ^114/110^Cd_bean–leaf_ values of −0.34‰ to −0.40‰ were obtained for Nacional cacao from two farms, whilst CCN-51 hybrid cacao from a third farm showed no fractionation within error (−0.08 ± 0.13‰). As such, further work to investigate whether Cd isotopes are indeed useful for tracing sources of Cd enrichments in soils and to inform genetic efforts to reduce the Cd burden of cocoa is indicated.

## Abstract 2

Cd isotope composition in cacao seems to be cultivar-specific whereas Cd in soil is probably due to tree litter recycling.

## Graphical abstract

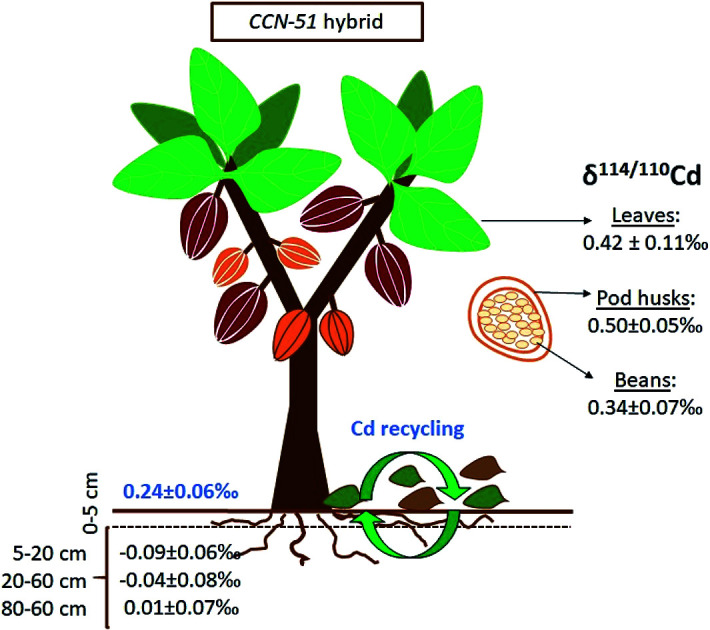

## 1. Introduction

The toxicity of Cd to animals and plants is well documented. For humans, Cd is highly toxic even at relatively low concentrations as it accumulates in the body over decades and is carcinogenic and damaging to kidneys and bones following chronic exposure.^1,2^ In response to concerns that cocoa often features relatively high Cd concentrations, the European Union implemented strict new upper limits on the Cd concentrations of cocoa products in 2019.^3^ This recent development has spurred research into the extent of Cd enrichment in cacao beans from different growing regions and the causes of the observed enrichments.

Such work has shown that there are large regional variations of Cd levels in cacao beans, whereby problematically high concentrations are particularly prominent in Latin America and some Asian countries.^4–13^ In these regions, the Cd concentrations of beans commonly exceed 0.6 to 0.8 mg kg^−1^,^14^ which is the approximate upper range for manufacturing cocoa products that are in accord with the new EU regulations.^8,15^ For example, a recent study of more than 500 farms in Ecuador revealed that nearly 50% of the cacao beans had Cd concentrations that exceeded 0.6 mg kg^−1^.^15^ For Ecuador and other affected countries, these thresholds may have grave economic consequences, whereby the numerous small-hold farmers and their families, which grow and harvest the cacao beans, might be most severely affected.

As Cd is a non-essential but toxic element, plants have developed specific sequestration strategies to cope with uptake of unwanted Cd from soils.^16,17^ In comparison to other food crops, however, cacao plants commonly take up significantly more Cd, such that they have been termed a Cd ‘accumulator’.^6,15^ Whilst the ultimate causes of Cd enrichment in beans remain unclear, the issue is of particular relevance, as the development of feasible mitigation strategies requires a better understanding of both natural and anthropogenic Cd sources and of the mechanisms that govern Cd uptake by cacao as well as transfer and accumulation of the element within the plants.

Most recent investigations of soil – cacao systems concur that high Cd levels in beans are correlated with high concentrations of mobile, and hence readily plant-available, Cd in soils.^5,6^ The interpretation of this observation differs, however. For example, particularly high Cd levels in the uppermost soil layers (<20 cm depth) were interpreted by some workers as a consequence of agricultural practices, such as the application of mineral P fertilizers,^6,8^ which often feature high Cd concentrations, and/or decomposition of cacao leaves and discarded pods, which are commonly left in plantations as an organic fertilizer.^8,12^ Other studies linked the high concentrations of plant-available Cd in soils to soil characteristics, such as pH and contents of Mn, Fe and Al oxyhydroxides, which affect the fraction of Cd that is readily mobile rather than tightly adsorbed to soil particles.^15,18^

Only limited understanding is available on the mechanisms that control Cd uptake by cacao from soils and the processes that take place within cacao, which are responsible for translocation of Cd from the roots to above-ground parts and the internal sequestration of the element. In particular, processes such as uptake into and retention in root cells, loading into the xylem, transfer from the xylem to the phloem and transport through phloem appear to play a key role in the Cd accumulation and distribution within cacao.^16,19–24^ It has also been proposed that Cd can be directly loaded from stems and branches into cacao beans without passing through leaves and that Cd is relocated into developing beans from pod husks.^25^

Previous studies on annual crops such as barley, potatoes and rice revealed clear cultivar-specific differences in Cd uptake and particularly root to shoot translocation, whereby the latter appears to be governed by the ability of plants to sequester Cd in roots with the aid of transporter proteins.^26–29^ Two recent investigations on cacao also revealed cultivar-specific differences in Cd handling but with contrasting results. One study found that cultivars primarily differ in the efficiency of Cd translocation from shoots to beans, due to differences in the efficiency of Cd transfer from xylem to phloem.^25^ In contrast, a second study revealed clear cultivar-related differences in soil to shoot translocation of Cd, that were attributed to differential Cd uptake and/or retention in the roots.^30^

There are only few previous Cd isotope investigations of plants and soil – plant systems. Such studies have already shown, however, that coupled Cd concentration and isotope data can be used to trace the origin of isotopically distinct Cd inputs, such as mineral P fertilizers, into soils.^31–35^ In addition, isotope data can provide constraints on the distinct cellular and molecular processes that are employed by plants for the uptake, internal transport and sequestration of Cd. In particular, recent publications demonstrated that wheat and barley display similar trends of Cd isotope fractionation^33^ and these differ significantly from the isotope systematics observed for Cd-tolerant and accumulator cultivars.^36^ As various genotypes of cacao differ in the extent to which Cd is enriched in the plants relative to soil and in how Cd is partitioned within the plants,^7,25,30^ such genotypes might also exhibit distinct Cd isotope signatures as a consequence of distinct pathways for Cd uptake, transport and sequestration.

In the following, results are presented from the first investigation that employed Cd isotope data for the study of soil – cacao systems. In detail, the study encompassed coupled Cd concentration and isotope analyses for 29 samples of soils, organic soil amendments and cacao plants from three farms in Ecuador. The data are applied to evaluate (i) whether they might provide constraints on the origin of any Cd enrichments in soils; and (ii) if different cacao cultivars exhibit distinct Cd isotope distributions, which may point to genetic differences in how the plants take up Cd and distribute the element between different organs.

## 2. Materials and methods

### 2.1. Study sites and samples

The samples for this study are from three small-scale cacao farms in Ecuador. Farm A is in the North Amazonian province of Sucumbíos, whilst Farms B and C are in the Pacific Coast province of Esmeraldas (Table 1 and ESI Fig. S1†). Although both provinces have similar average annual temperatures (∼25 °C) and total annual rainfall (>3000 mm), precipitation is evenly-distributed in the North Amazon region but seasonal near the Pacific Ocean.^37,38^ All three farms do not apply irrigation water and they follow organic practice, with no use of mineral P fertilizers, chemical pesticides or herbicides. Whilst assurances were provided by the farmers that organic practices had been followed for several years at all sites, independent verification of this information is not possible. Farm A is a cacao monoculture plantation and one of the two sites investigated at this farm (Site A-1) is located on an island in the Aguarico River. At Farm B, the cacao grows alongside timber trees, whilst cacao, timber, banana and avocado trees are grown together at Farm C (Table 1).

**Table 1:** Information on the sampling sites and the cacao plants and soils at these locations

Sampling site	A-1	A-2	B	C-a	C-b
GPS coordinates	0°1′41.08′′ N	0°1′41.08′′ N	0°29′50.04′′ N	0°45′55.48′′ N	0°45′55.48′′ N
	76°36′36.82′′ E	76°36′36.82′′ E	79°51′8.41′′ E	79°56′5.06′′ E	79°56′5.06′′ E
Province	Sucumbíos	Sucumbíos	Esmeraldas	Esmeraldas	Esmeraldas
Cacao cultivar	Nacional hybrid	CCN-51 hybrid	Nacional	Nacional	Nacional
Age of trees	Unknown	Unknown	6 years	10 years	20 years
Culture system	Cacao monoculture	Cacao monoculture	Cacao & timber trees	Cacao, banana, avocado, timber trees	Cacao, banana, avocado, timber trees
Soil type	Andisol	Ultisol	Inceptisol	Mollisol	Mollisol
Soil pH^a^	6.78	6.52	6.30	7.14	7.08
Soil CEC (cmol kg^−1^)^a^	37.0	39.8	7.7	2.9	1.7
Organic soil amendments^b^	Chicken manure, (tree litter)	Chicken manure (tree litter)	Tree litter (cattle manure)	Tree litter (cattle manure)	Tree litter (cattle manure)

a The pH and CEC values for 0–20 cm depth were calculated for Sites A-1 and A-2 (from individual data for 0–5 cm and 5–20 cm layers) and directly measured for soils from Farms B and C. The detailed soil data for Sites A-1 and A-2 are available in Table S4 of the ESI.

b Organic soil amendments in parentheses are used in the farms but were not sampled and analyzed in this study.

There are two main Ecuadorian cacao varieties. The Nacional cultivar produces fine-flavour specialty cocoa whilst CCN-51 is a high-yield cultivar that is resistant to fungal diseases but provides cocoa of lower aromatic quality.^39,40^ In most farms, various hybrids of these two varieties are cultivated. In the current study, the cacao samples were assigned to three distinct cultivars, based on phenotype and information supplied by the farmers. The two sites at Farm A are used to cultivate different cacao phenotypes, which were termed Nacional hybrid and CCN-51 hybrid cacao plants, respectively, by the farmers. The samples from Farm B and two sites at Farm C were provided by the French Cooperative ‘Ethiquable’ as part of a larger study, which investigates the effects of agricultural practices and cacao processing on Cd concentrations in beans. The cacao from these sites is phenotypically similar and classified as a relatively pure Nacional cultivar by the cooperative. Similar sampling methods were employed at all sites, which followed established practices^8^ and are further detailed in the ESI.†

From Farm A, mature cacao leaves as well as the husks and beans of mature cacao pods were sampled: Nacional hybrid cacao at Site A-1 and a CCN-51 hybrid cacao at Site A-2 (Table 1). Cocoa liquor produced from these two varieties of cacao was also analysed (see ESI for details†). The soil samples of Farm A were collected at depths of 0–5 cm, 5–20 cm, 20–60 cm, and 60–80 cm. Farm-produced chicken manure that was used as an organic soil amendment at Sites A-1 and A-2 was collected at both locations (Table 1).

The samples from Farm B and two proximal sites at Farm C encompass cacao beans and leaves from a 6 year old Nacional tree at Farm B and two Nacional trees with ages of 10 years (Site C-a) and 20 years (Site C-b) at Farm C (Table 1). The beans from the C-a and C-b sites were combined by the cooperative to prepare a single mixed cacao bean sample for a quality assay, and this was subsequently made available for this investigation. In addition, 0–20 cm soil samples were taken in the vicinity of the sampled trees at Farms B and C (Table 1), as most of the cacao roots are located at this depth.^41,42^ The natural tree litter, which was used as organic fertilizer in addition to cattle manure at Farms B and C, was collected from underneath the sampled cacao trees (see ESI for details†).

### 2.2. Initial sample preparation and physicochemical analyses of soils

At the GET Laboratory in Toulouse, all samples were first air and then oven-dried at 45 °C. The samples were subsequently homogenized using a vibrating cup mill with a steel grinding set and agate discs (soils, chicken manure, litter, cacao leaves, pod husks) or in an agate mortar with liquid nitrogen (cacao beans with shells, cocoa liquor).

The physicochemical characterisation of the soils in Toulouse included (i) measurement of soil pH with a Metrohm 744 pH meter on a 1 : 5 mixture of ground soil in water and (ii) determination of cation exchange capacity (CEC) by spectrocolorimetry using cobalt hexamine trichloride.^43^

### 2.3. Determination of Cd concentrations and isotope compositions

#### 2.3.1. Sample digestion and initial Cd concentration measurements

Samples of about 100 to 200 mg were digested in the GET clean room laboratory in Toulouse using Merck Suprapur and bi-distilled reagents (see ESI for details†). A minor solution aliquot of the sample solutions was employed for a preliminary determination of Cd concentrations in the Platform AETE-ISO, OSU OREME at the University of Montpellier (France), using a Thermo Scientific iCAP Q-ICP-MS instrument. These analyses later enabled optimal double-spiking for the Cd isotope measurements. Standard reference materials were used to validate the digestion methods for soil and plant samples (Table S2 of ESI†).

#### 2.3.2. Cd separation and Cd isotope measurements

Subsequent analytical work, including sample preparation in a clean room laboratory and the mass spectrometric analyses was carried out in the MAGIC Laboratories at Imperial College London. The employed methods were previously described in detail,^31,44^ but key information is given here and in the ESI.† A ^111^Cd–^113^Cd Cd double spike was first added to and equilibrated with the main sample solution aliquot to obtain ratios of spike- to sample-derived Cd (S/N) of about 1.1. The Cd was then isolated from the sample matrix and purified using anion exchange and extraction chromatography, followed by liquid–liquid extraction.

The isotopic analyses were performed with a Nu Instruments Nu Plasma HR MC-ICP-MS (multiple collector inductively coupled mass spectrometer), using an Aridus II desolvation system with a PFA nebulizer (CETAC Technologies). The sample analyses were interspersed between runs of spiked solutions of the NIST SRM 3108 Cd isotope reference material,^45^ which were measured at dilutions and S/N values comparable to the samples. The isotopic compositions of the samples are reported relative to the NIST SRM 3108 Cd standard:
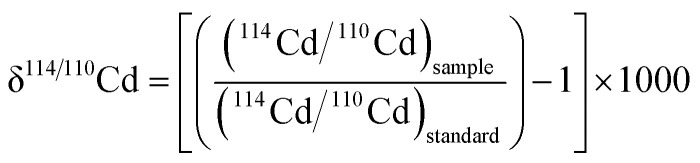


The quoted analytical precision of the δ^114/110^Cd data (Table 2) was estimated from the repeatability of the bracketing analyses of matching NIST 3108 Cd–Cd DS mixtures, which was typically between 0.05‰ and 0.10‰ (2sd). The isotopic data obtained in the sample runs were also processed using the isotope dilution technique to determine the final Cd concentrations of the samples.

**Table 2:** Cadmium concentrations and isotope compositions (as δ^114/110^Cd)^a^ for soils, organic soil amendments, as well as cacao leaves, pod husks and beans, and cocoa liquor that were obtained from five sites at three farms in Ecuador

Sampling site	A-1, Nacional hybrid cacao		A-2, CCN-51 hybrid cacao		B, Nacional cacao		C-a, Nacional cacao		C-b, Nacional cacao	
	Cd (mg kg^−1^)	δ^114/110^Cd (‰)	Cd (mg kg^−1^)	δ^114/110^Cd (‰)	Cd (mg kg^−1^)	δ^114/110^Cd (‰)	Cd (mg kg^−1^)	δ^114/110^Cd (‰)	Cd (mg kg^−1^)	δ^114/110^Cd (‰)
**Soil samples and organic soil amendments**										
0–5 cm	0.515	0.19 ± 0.04	1.10	0.24 ± 0.06	—	—	—	—	—	—
5–20 cm	0.260	−0.07 ± 0.09	0.345	−0.09 ± 0.06	—	—	—	—	—	—
0–20 cm	0.324^b^	0.01 ± 0.08^b^	0.533^b^	0.04 ± 0.06^b^	0.384	0.10 ± 0.05	1.60	−0.09 ± 0.05	0.809	−0.03 ± 0.05
20–60 cm	0.395	−0.09 ± 0.06	0.253	−0.04 ± 0.08	—	—	—	—	—	—
60–80 cm	0.167	−0.27 ± 0.10	0.734	0.01 ± 0.07	—	—	—	—	—	—
Chicken manure	0.271	−0.09 ± 0.05	0.273	0.11 ± 0.07	—	—	—	—	—	—
Tree litter	—	—	—	—	3.84	0.28 ± 0.08	6.23	0.19 ± 0.04	1.15	0.29 ± 0.04

**Cacao trees and cocoa products**										
Leaves	2.12	0.42 ± 0.04	2.38	0.42 ± 0.11	3.63	0.31 ± 0.05	3.06	0.30 ± 0.05	1.95	0.28 ± 0.06
Pod husks	0.99	0.53 ± 0.05	2.14	0.50 ± 0.05	—	—	—	—	—	—
Beans	1.26	0.18 ± 0.05	2.17	0.34 ± 0.07	3.38	−0.02 ± 0.05	3.92^c^	−0.11 ± 0.05^c^	3.92^c^	−0.11 ± 0.05^c^
Cocoa liquor	1.44	0.10 ± 0.04	3.95	0.33 ± 0.05	—	—	—	—	—	—

a The quoted errors for the δ^114/110^Cd data denote the 2sd mass spectrometric reproducibility of the isotopic measurements.

b Weighted mean calculated from measured results for 0–5 cm and 5–20 cm soil layers.

c The cacao beans from Sites C-a and C-b were mixed and analyzed together; hence only a single result is given (see ESI for details).

Procedural blanks processed alongside the samples were about 50 pg Cd during the study. This corresponds to a contribution of less than 0.1% to the total Cd present in the samples. The blank was subtracted from the measured Cd concentrations but no corrections were applied to δ^114/110^Cd data. Quality control and standard reference materials were analyzed to validate the accuracy and precision of the measurements. These results are in good agreement with independent reference values (Table S3 of ESI†).

For the comparison of Cd isotope compositions, the apparent isotope fractionation between two samples or reservoirs was calculated as:Δ^114/110^Cd_A–B_ = δ^114/110^Cd_A_ − δ^114/110^Cd_B_where A and B denote the two Cd reservoirs.

## 3. Results

### 3.1. Soil taxonomy and properties

At Farm A, the young volcanic andisols of Site A-1 differ from the older, more intensely weathered ultisols at Site A-2. Both, however, display a narrow range of pH values from about 6.3 to 7.0 and CECs of between 30 and 53 cmol kg^−1^ (Tables 1 and S4 of ESI†). The relatively young inceptisol at Farm B was also slightly acidic with pH ≈ 6.3 but the CEC was significantly lower at about 8 cmol kg^−1^. The base-rich mollisol of Farm C had the highest pH value of about 7, combined with the lowest CEC of between 1.7 and 2.9 cmol kg^−1^ (Tables 1 and S4 of ESI†).

### 3.2. Cd concentrations and isotope compositions of soils

**Fig. 1:**
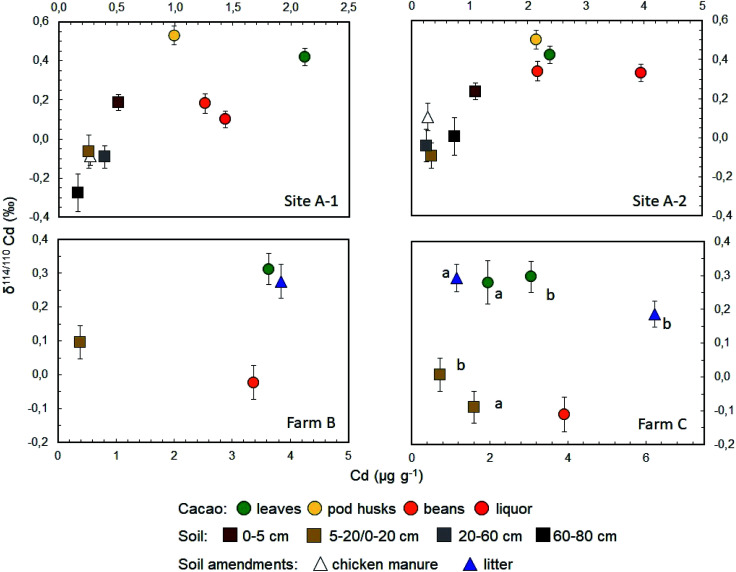
Plots of Cd isotope data (as δ^114/110^Cd) *versus* Cd concentrations for the leaves, pod husks, beans and liquor of cacao plants as well as for soils from different depths and organic soil amendments (chicken manure, leaf litter). The different panels show results for samples from (a) Site A-1, (b) Site A-2, (c) Farm B and (d) Farm C (samples from locations C-a and C-b are denoted by letters “a” and “b”, respectively). Nacional hybrid and CCN-51 hybrid cacao cultivars are cultured at Sites A-1 and A-2 of Farm A, respectively, whilst relatively pure Nacional cultivars are grown at Farms B and C. The error bars denote the mass spectrometric reproducibility of the Cd isotope data.

**Fig. 2:**
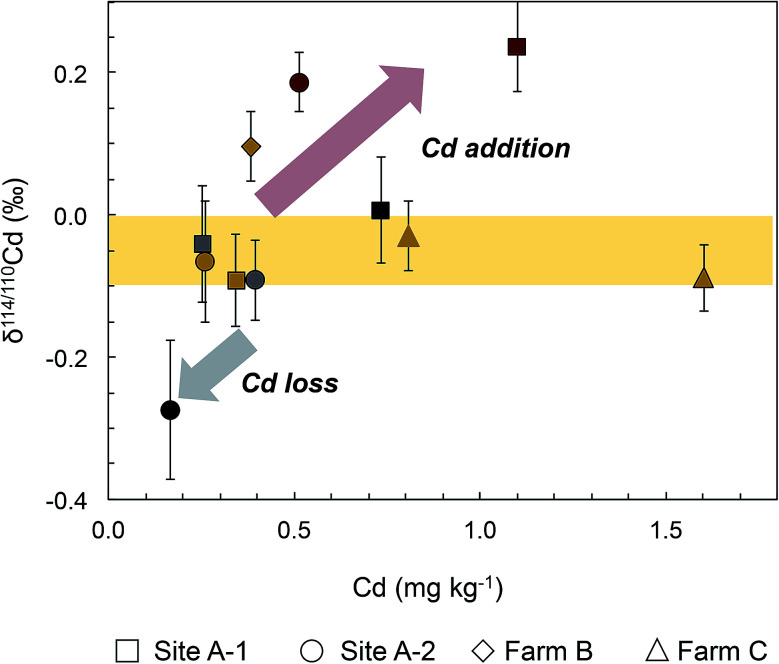
Plot of Cd isotope data (as δ^114/110^Cd) *versus* Cd concentrations for the soil samples analyzed in this study. Most soils have a narrow range of δ^114/110^Cd from about 0‰ to −0.1‰, as indicated by the yellow field. The arrows show schematically how Cd loss and Cd addition (from decomposing plant material) affected soil samples from Farms A and B. The symbol colors are the same as in figure, but different shapes denote the sampling sites A-1 (circles), A-2 (squares), Farm B (diamonds) and Farm C (triangles; symbols with and without black outlines denote samples from locations C-a and C-b, respectively). The error bars denote the mass spectrometric reproducibility of the Cd isotope data.

The Cd concentrations of the soils vary by roughly an order of magnitude, from 0.167 mg kg^−1^ at 60–80 cm depth of Site A-1 to 1.60 mg kg^−1^ at 0–20 cm of location C-a (Fig. 1, 2 and Table 2). A marked variability in concentrations is thereby seen both within the soil profiles of sites A-1 and A-2 (by about a factor of ×3 to ×4) as well as between the proximal sites A-1, A-2 and C-a, C-b. This variability may reflect relatively heterogeneous Cd concentrations even at small length scales in the field or the relatively small sample aliquots (of 100–200 mg) that were digested for analysis.

In contrast to the concentrations, the Cd isotope compositions of most soil samples show only limited variability (Fig. 1, 2 and Table 2). In detail, 6 of the 10 soil samples have δ^114/110^Cd values of between 0.0‰ and −0.1‰ and are identical within analytical error. One of the remaining samples is isotopically light with δ^114/110^Cd = −0.27‰ whilst the other three are topsoils with δ^114/110^Cd values of between 0.10‰ and 0.24‰ (Fig. 1, 2 and Table 2).

### 3.3. Cd concentrations and isotope compositions of cacao plant samples

All cacao leaves are enriched in Cd relative to the soils, with concentrations between 1.95 to 3.63 mg kg^−1^. They are also characterized by nearly identical heavy Cd isotope compositions with δ^114/110^Cd values from about 0.3‰ to 0.4‰ (Fig. 1 and Table 2). The two cacao pod husks also have variable Cd concentrations akin to the leaves, with 0.99 to 2.14 mg kg^−1^ Cd, and similar or slightly higher δ^114/110^Cd of about 0.5‰ (Fig. 1 and Table 2).

The Cd concentrations of the four cacao bean samples resemble the leaves, with concentrations of 1.26 to 3.92 mg kg^−1^ but the Cd isotope compositions are more variable (Fig. 1 and Table 2). In detail, the two bean samples from Farm A have positive δ^114/110^Cd values that are distinct, considering the analytical errors, at 0.18 ± 0.05‰ and 0.34 ± 0.07‰. In contrast, the bean samples from Farms B and C display lighter Cd isotope compositions, with δ^114/110^Cd values that are identical, within uncertainty, at −0.02 ± 0.05‰ and −0.11 ± 0.05‰.

### 3.4. Cd concentrations and isotope compositions of additional samples

Two samples of chicken manure from Farm A, which was used as organic fertilizer, have relatively low Cd concentrations of about 0.27 mg kg^−1^ and δ^114/110^Cd values similar to the soils, with results of −0.09‰ and 0.11‰ (Fig. 1 and Table 2). The tree litter from Farms B and C, displays Cd systematics that resemble but are slightly more variable than the fresh cacao leaves. In detail, the Cd concentrations vary between 1.15 to 6.23 mg kg^−1^ whilst the δ^114/110^Cd values range from 0.19‰ to 0.29‰. Finally, the cacao liquors from Farm A have higher Cd concentrations than the beans from the same sampling sites but essentially identical δ^114/110^Cd values (Fig. 1 and Table 2).

## 4. Discussion

Compared to most other recent studies of Cd concentrations in cacao and soil – cacao systems, the current investigation features results for a relatively small number of samples but these data are coupled with novel Cd isotope results. As such, the discussion primarily focuses on the latter results.

### 4.1. Cd distribution and isotope fractionation in the soils

The variable Cd concentrations of the soils are in accord with previously reported total Cd concentrations for soils from different locations in Ecuador.^5,8,15^ Notably, the new soil Cd data show no systematic variation with soil type or soil properties and, for the soil profiles from Farm A, with depth.

The eight soil samples from the two profiles of Farm A are, however, in accord with a rough positive correlation of δ^114/110^Cd with Cd concentration, and two of the three 0–20 cm soil samples from Farms B and C are also in accord with such a trend (Fig. 2). The small dataset obtained here does not allow a rigorous evaluation of the inferred correlation but some interpretations can be developed. Notably, the majority of soils cluster at Cd concentrations of 0.2 to 0.8 mg kg^−1^ and they have δ^114/110^Cd values of between −0.1‰ and 0.0‰, similar to the estimated composition of the upper continental crust (with δ^114/110^Cd ≈ 0).^46,47^ Compared to this, the 60–80 cm soil from site A-2 has a lower Cd concentration and lower δ^114/110^Cd of −0.27 ± 0.10‰ (Fig. 2 and Table 2). The latter observation is most readily explained by loss of Cd from the solid soils to pore waters by leaching during weathering, as soil solutions are expected to be enriched in heavy Cd isotopes relative to the residual solid.^34,48,49^ The dissolved Cd is subsequently removed from the soils by drainage of the pore waters and Cd uptake by plants.^34^

The scattered δ^114/110^Cd *versus* Cd trend of the soil samples is, furthermore, governed by the relatively high Cd concentrations and δ^114/110^Cd values (of 0.19‰ to 0.24‰) for the two 0–5 cm topsoils from Farm A. In addition, a relatively high δ^114/110^Cd of 0.10‰ is also observed for the 0–20 cm soil of Farm B (Fig. 2 and Table 2). As the two 0–5 cm soils from Sites A-1 and A-2 have Cd concentrations that clearly exceed the deeper soil layers, it is likely that they record Cd addition to the soil surface. The relatively high δ^114/110^Cd of the Cd-rich topsoils is, furthermore, in accord with the interpretation that the added Cd is derived from plant material that decomposed at the surface whilst other possible sources of added Cd are unlikely. The latter conclusion is discussed in the following for (i) chicken manure, (ii) aerosols, (iii) river sediments and (iv) mineral P fertilizers as plausible Cd sources.

(i) Chicken manure, which is added to the soils at Farm A for fertilization, has Cd concentrations and δ^114/110^Cd values that are lower compared to the topsoils (Fig. 2 and Table 2). As such, it likely has only a limited impact on topsoil Cd budgets.

(ii) A recent isotopic study investigated diverse marine aerosols with Cd budgets that varied in origin between primarily natural (most likely from mineral dust) to dominantly anthropogenic. Despite this, 11 of the 12 investigated aerosols had very similar δ^114/110^Cd values of between −0.1‰ and 0.1‰. One particularly Cd-rich sample, most likely from anthropogenic sources, featured δ^114/110^Cd ≈ −0.5‰.^50^ The latter result is in accord with data for fumes and ash from metal smelting operations, which are also enriched in light Cd isotopes, most likely due to preferential release of isotopically light Cd in high-temperature processes.^51–54^ Together, this suggests that atmospheric additions of Cd, for example from volcanic emanations in the Andes, forest fires or the slash and burn farming practices that are common in the Amazon region^55^ and industrial sources (*e.g.*, oil refinery or shipping port in Esmeraldas), are probably not responsible for the enrichment of isotopically heavy Cd in the topsoils of Farms A and B.

(iii) It is also unlikely that the topsoil enrichment of Cd results from riverborne sediments, which might have been emplaced during flooding by the river at Farm A. This follows from the inference that such sediments will likely feature δ^114/110^Cd values similar to or slightly lower than average upper continental crust, for which data are available from a few studies.^46,47^ Support for this inference, is provided by the limited variability of Cd isotope compositions found so far in silicate rocks and clastic sediments,^46,56^ as well as the observation that silicate weathering appears to deplete residual rocks in isotopically heavy Cd (Fig. 2; ref. 31, 33, 34 and 48). Given an average upper continental crust with δ^114/110^Cd = −0.01‰ ± 0.04‰, river sediments with similar or lower δ^114/110^Cd values are unable to account for the heavy Cd isotope compositions of the two Farm A topsoils (Fig. 2 and Table 2).

(iv) Mineral P fertilizers can feature high Cd contents but are an improbable source for the observed topsoil Cd enrichment as such fertilizers were not used at any of the farms for the past years. Furthermore, Cd isotope data for numerous different mineral P fertilizers reveal that the great majority are characterized by δ^114/110^Cd values of less than 0.20‰.^33–35^ Use of such fertilizers in the more distant past is thus unlikely to have caused the observed high δ^114/110^Cd = 0.24‰ for the 0–5 cm topsoil at Site A-2 (Fig. 2 and Table 2).

Given these results, it is most likely that the enrichment of Cd characterized by positive δ^114/110^Cd values in the topsoils of Farms A and B are due to addition of the element from decomposing cacao tree litter. This conclusion is reasonable as tree litter and cacao leaves feature both high Cd concentrations and relatively heavy Cd isotope compositions, with δ^114/110^Cd values of about 0.2‰ to 0.3‰ (Fig. 1 and Table 2). Notably, it is common practice to leave pod husks and leaves in cacao fields after harvest for organic fertilization and it was previously suggested that the rapid decomposition of such material in tropical conditions may represent an important source of Cd to the uppermost soil layer.^6,8^ The interpretation is further supported by data for birch leaves from Sweden, which were also found to have ubiquitously heavy Cd isotope compositions with a mean δ^114/110^Cd of 0.70 ± 0.20‰ (1sd, *n* = 83).^57^

Of further interest in this context are recent detailed studies of soil-wheat agricultural systems in Switzerland, which also identified Cd enrichments characterized by heavy isotope compositions in topsoils.^31,33^ Based on a detailed soil mass balance, it was concluded that these Cd enrichments are unlikely to be of recent origin but rather reflect long-term ‘plant-pumping’, whereby uptake of Cd by plants and trees, subsequent decay of the organic matter with relatively high δ^114/110^Cd and release of this Cd into the topsoil over long timescales is responsible for the Cd enrichment. In the absence of a detailed soil mass balance for the cacao sites of this study, it is therefore uncertain whether the observed topsoil Cd enrichments have a long-term origin, which predates cacao cultivation or reflect recent agricultural practices, such as the use of leaf litter as a soil supplement. This question can be addressed but this would require a detailed investigation of the Cd budget and mass balance of the soils, including relevant input and output fluxes of the element.

### 4.2. Cd distribution and isotope fractionation in the cacao tissues

For all investigated cacao samples, the Cd concentrations significantly exceed those observed for the soils from the same location. Using the measured or calculated Cd soil concentrations for 0–20 cm as a reference (Table 2), Cd shows enrichment factors of between about ×2 to ×10 in both leaves and beans, whereby the leaf and bean enrichment factors are roughly similar for all 4 sites (Table 3). The observed Cd concentrations and enrichment factors are in accord with data from previous investigations of cacao plantations in Ecuador, which considered a larger number of samples and locations.^8,15^

**Table 3:** Enrichment factors for Cd determined for various parts of cacao trees and associated Cd isotope fractionation^a^

Sampling site	A-1	A-2	B	C^b^
**Cd enrichment factors [Cd] in A/[Cd] in B**				
Leaves/soil 0–20 cm	6.5	4.5	9.5	2.1
Beans/soil 0–20 cm	3.9	4.1	8.8	3.2
Beans/leaves	0.6	0.9	0.9	1.6

**Apparent Cd isotope fractionation Δ** ^**114/110**^ **Cd**_**A–B**_				
Leaves–soil 0–20 cm	0.41 ± 0.09	0.38 ± 0.13	0.22 ± 0.07	0.35 ± 0.07
Beans–soil 0–20 cm	0.17 ± 0.08	0.30 ± 0.10	−0.12 ± 0.07	−0.05 ± 0.07
Beans–leaves	−0.24 ± 0.07	−0.08 ± 0.13	−0.34 ± 0.07	−0.40 ± 0.08

a The quoted errors for Δ^114/110^Cd are derived by propagating the uncertainties of the individual δ^114/110^Cd results (Table 2; see ESI for details).

b The Cd enrichment and apparent isotope fractionations for Farm C samples were calculated using the weighted mean Cd concentrations and isotope compositions from Sites C-a and C-b. Only a single mixed bean sample (encompassing beans from C-a and C-b) was analyzed.

For the samples analysed here, the observed Cd enrichment factors show no systematic variation with cultivar, as the CCN-51 hybrids of Site A-2 reveal Cd enrichments that are intermediate to those observed for the Nacional hybrids from Site A-1 and the Nacional cacao of Farms B and C (Table 3). Instead, it appears that the enrichment of Cd in the cacao plants is, at least in part, governed by soil pH, which is known to have an impact on the extent to which soil-bound Cd is available to plants.^5,8,15^ In detail, the cacao leaves and beans of Farm C, with the highest observed soil pH of about 7.1, show the lowest Cd enrichment factors of about ×2 to ×3 (Table 3). In contrast, the highest enrichment factors of about ×9 to ×10 were found at Farm B, where the 0–20 cm soil has the lowest pH value of 6.3. Finally, intermediate Cd enrichments of about ×2 to ×6 were observed for the cacao plants from Farm A, where the soils have intermediate pH values of about 6.5 to 6.8. This conclusion, however, should be viewed with caution, due to the small number of samples investigated here and the limited variation in soil pH.

The Cd isotope compositions of the cacao tissues also reveal systematic distributions. In particular, the cacao leaves and pod husks consistently display Cd isotope compositions, which are heavier compared to the soil from the same location (Fig. 1). This observation is particularly clear if data for the 0–5 cm soils from Sites A-1 and A-2 are disregarded, as these samples most likely have higher δ^114/110^Cd than deeper soils due to admixture of leaf-derived Cd. In the following, measured or calculated 0–20 cm soil samples are therefore used for reference (Table 2), in accord with the observation that most cacao roots are situated at this depth.^41,42^ Using these data, the isotope fractionation between the cacao leaves and soils from Sites A1, A2 and farm C is identical with Δ^114/110^Cd_leaf–soil_ of between 0.35 ± 0.07‰ to 0.41 ± 0.09‰, and a similar value of 0.22 ± 0.07 is observed for Farm B.

An enrichment of isotopically heavy Cd relative to soils is also seen for the cacao pod husks from Sites A-1 and A-2, which have δ^114/110^Cd values that agree with the leaves to within less than 0.1‰ (Fig. 1 and Table 2). As such, the transfer of Cd from the soils to leaves and pods is associated with essentially the same isotope fractionation. Similarly, tree litter from sites B and C-b displays Cd isotope compositions that are identical to mature leaves picked from trees of the same site (Fig. 1 and Table 2). This suggests that the initial decomposition of the leaves is not associated with any substantial Cd isotope fractionation, in accord with the previous conclusion that Cd derived from decomposing tree litter delivers isotopically heavy Cd to topsoils. Notably, these results contrast with Zn isotope data acquired for *Typha latifolia*, as heavy Zn isotopes were found to be enriched in mature and decaying relative to fresh leaves.^58^ These contrasting observations may reflect that Zn, as an essential micronutrient, is intensively translocated within plants *via* the phloem whilst such translocation is expected to be less important for non-essential Cd.^59^

The cacao beans feature more complex Cd isotope systematics. At Farms B and C, the Nacional beans display the lowest δ^114/110^Cd coupled with negative Δ^114/110^Cd_bean–leaf_ values of −0.34‰ and −0.40 (Tables 2 and 3). In contrast, the CCN-51 hybrid beans from Site A-2 harbor the highest δ^114/110^Cd and no resolvable Cd isotope fractionation between beans and leaves with Δ^114/110^Cd_bean–leaf_ ≈ −0.08 ± 0.13. Finally, the Nacional hybrid beans of Site A-1 have δ^114/110^Cd, Δ^114/110^Cd_bean–leaf_ and Δ^114/110^Cd_bean–soil_ values that are intermediate to those recorded at Sites A1 and Farms B, C (Tables 2 and 3).

Notably, the cacao leaves and beans from Farms B and C display identical δ^114/110^Cd values, within error, and identical leaf–soil and bean–leaf Cd isotope fractionation (Fig. 1, Tables 2 and 3). As the trees of these farms are all from the Nacional cultivar but vary in age between 6 years and 10 to 20 years, the results suggest that tree age may not have a substantial impact on the uptake and translocation of Cd by cacao plants.

### 4.3. Cd isotope fractionation in other plants

It is of interest to compare the Cd isotope data obtained for cacao with results acquired for other plants in recent studies. These comparative data are, however, limited to the cereals wheat and barley as well as perennial Cd-tolerant and accumulator plants. Notably, the more complex architecture and physiology of cacao trees^60,61^ is expected to be associated with distinct internal Cd fluxes between the plant organs, whilst different molecular mechanisms of Cd transport and sequestration might also be relevant.

The enrichment of isotopically heavy Cd in the leaves, pods and beans of cacao is in accord with observations made for wheat, whereby the straw and grains of the latter were found to have higher δ^114/110^Cd values than the soils.^31^ In part, this isotopic difference reflects that the plant-available (PA) Cd pool of soils typically appears to be characterized by higher δ^114/110^Cd than bulk soils, with a fractionation factor of up to Δ^114/110^Cd_PA–soil_ ≈ 0.5‰.^31,33^ As such, bulk wheat plants have Cd isotope compositions that closely resemble the plant-available Cd of soils but with significant internal Cd isotope fractionation, whereby successively higher δ^114/110^Cd values are observed for the roots, the straw and the grains. Amongst these compartments, only the grains are consistently enriched in isotopically heavy Cd relative the plant-available soil Cd, with an apparent fractionation of Δ^114/110^Cd_PA–grain_ ≈ 0.1–0.5‰.^31,33^ In contrast, the roots and straw of wheat have Cd isotope compositions that are lighter than or roughly similar to the plant-available Cd of soils, respectively.

Different systematics were identified for the Cd-tolerant plant *Ricinus communis* L. and the Cd accumulator *Solanum nigrum* L. In both cases, the plants were enriched in light Cd isotopes compared to the hydroponic nutrient solution, and the largest fractionations were observed between the roots and the hydroponic solution, with Δ^114/110^Cd_root–solution_ ≈ −0.4‰.^36^ However, clear differences in isotope fractionation patterns were observed within the plants. In detail, the Cd accumulator displayed only little or no isotope fractionation between the roots, stems and leaves. In contrast, the Cd-tolerant *R. communis* revealed variable isotope fractionation between stems and roots but a consistent enrichment of isotopically light Cd in the leaves relative to stems, with Δ^114/110^Cd_leaf–stem_ of between −0.04‰ and −0.33‰.

### 4.4. Cd isotope fractionation between cacao leaves and soil

Considering the literature results, it is likely that the relatively consistent isotope fractionation about 0.2‰ to 0.4‰ observed between cacao leaves and the bulk soil of all four sites (Table 3) reflects a combination of (i) enrichment of isotopically heavy Cd in the plant-available Cd relative to the total Cd budget of the soils as well as (ii) sequestration of isotopically light Cd in the cacao roots and thus selective translocation of heavy Cd isotopes to the shoots. This conclusion is reasonable even though no data were acquired here for roots or soil solutions, as enrichments of (i) isotopically heavy Cd in soil solutions or leachates relative to bulk soils and (ii) isotopically light Cd in roots relative to the plant-available Cd seem to be consistent characteristics of plant-soil systems investigated to date.^31,33^

Given these constraints, the fairly consistent Δ^114/110^Cd_leaf–soil_ values observed here most likely reflect isotopically similar pools of plant-available Cd in soils, which are a consequence of the relatively similar mean δ^114/110^Cd and pH values observed for the soil profiles of all four sites (Tables 1 and 2). In addition, they also require that the sequestration of isotopically light Cd in the roots produces a residual, isotopically heavier pool of Cd in the plants which, after translocation, is incorporated in leaves that have consistent δ^114/110^Cd values of between 0.28‰ and 0.42‰ (Fig. 1 and Table 2). The observation of similar leaf Cd isotope compositions and Δ^114/110^Cd_leaf–soil_ values may thus indicate that the three studied cacao cultivars employ similar molecular strategies of Cd sequestration in the roots. Notably, this mechanism might resemble the Cd sequestration processes that are employed by wheat plants. This conclusion follows from the finding that wheat cultivated on two soils with Cd concentrations and pH values similar to those seen here (at 0–20 cm) displayed Cd isotope fractionations between soils and stems (Δ^114/110^Cd_stem–soil_) of 0.27‰ and 0.45‰.^33^ For wheat, this isotope fractionation was inferred to reflect mechanisms to avoid Cd accumulation in grains, which encompass sequestration of isotopically light Cd in roots.

### 4.5. Cd isotope fractionation between cacao beans and leaves

Whilst the Cd isotope fractionation between leaves and soil is similar for the cacao of all four sites there are clear differences in the bean–leaf fractionation Δ^114/110^Cd_bean–leaf_ observed for the plants (Tables 2 and 3). Even though the sample numbers are limited, it is conceivable that these differences in Cd isotope compositions and fractionations may be linked to distinct cacao cultivars, as different cultivars may feature distinct molecular pathways by which Cd is partitioned and sequestered within the plants. This tentative inference is in accord with previous elemental studies, which revealed significant differences in the partitioning of Cd between plant organs for different cultivars of cacao,^7,30^ and other crops.^26–29^ Also notable is that the enrichment of light Cd isotopes in beans of the relatively pure Nacional cultivar (Tables 2 and 3) is in accord with observations made for Cd-tolerant and Cd accumulator plants, as these also revealed an enrichment of light Cd isotopes in stems and leaves relative to the hydroponic solution in which they were cultured.^62^ The latter observation was thereby linked to the particular molecular mechanisms employed by these plants to cope with high levels of toxic Cd. As such, it would be of interest to interrogate the tentative inference of the current study in a more thorough follow-up investigation.

## 5. Conclusions

Results are presented from a pilot investigation that employed coupled Cd isotope and concentration measurements to study the distribution and cycling of Cd in soil – cacao systems of Ecuador. The results provide two particularly interesting observations.

First, most of the soil samples from 0 to 80 cm depth have relatively uniform δ^114/110^Cd of about −0.1‰ to 0‰. However, one sample from 60–80 cm is depleted in Cd and isotopically lighter, in accord with a weathering signature. Furthermore, two 0–5 cm topsoils have high Cd concentrations coupled with heavy Cd isotope compositions of δ^114/110^Cd ≈ 0.2%. This distinct isotopic signature suggests that the enrichment is unlikely to be derived from organic or mineral P fertilizers, natural or anthropogenic atmospheric contributions or sediments from a nearby stream. Rather, the Cd additions are probably sourced from decomposing tree litter. Further study is required, however, to ascertain whether the Cd enrichments reflects recent use of tree litter as fertilizer in the cacao plantations or natural accumulation of Cd from decomposing tree material over a much longer timescale.

Second, the beans of the three investigated cacao cultivars appear to show differences in Cd isotope signatures. In detail, beans from CCN-51 hybrid plants show no resolvable Cd isotope fractionation relative to the leaves whilst the relatively pure Nacional cultivar has beans that display lower δ^114/110^Cd than the leaves, with Δ^114/110^Cd_bean–leaf_ values of −0.34‰ and −0.40‰. As such, the data may suggest that the Nacional and CCN-51 cultivars differ in the molecular mechanisms that are employed for the translocation and sequestration of Cd between cacao leaves, pods and beans.

In summary, these results suggest that further, more comprehensive, coupled Cd isotope and concentration studies should be carried out to confirm whether such analyses indeed provide useful constraints on the origin and cycling of Cd in soil – cacao systems and the partitioning of Cd within cacao plants. In particular, the isotopic data may be of utility for molecular and genetic studies,^63^ which intend to identify the transporter proteins that govern Cd accumulation and partitioning in cacao plants, as well as for breeding efforts and the development of farming practices that aim to reduce the relatively high Cd concentrations of cacao beans grown on Cd-rich soils.

## Conflicts of interest

There are no conflicts to declare.

## Supplementary Material

RA-009-C9RA05516A-s001
