# Supplementary material for: Cadmium isotope fractionation in the soil – cacao systems of Ecuador: a pilot field study
Source: RSC Adv. 2019 Oct 23;9(58):34011–22. doi: 10.1039/c9ra05516a (PMC9073709; doi:10.1039/c9ra05516a)
Supplement: RA-009-C9RA05516A-s001 [file RA-009-C9RA05516A-s001.pdf]

Electronic Supplementary Material (ESI) for RSC Advances.
This journal is © The Royal Society of Chemistry 2019

Electronic Supplementary Information to:

Cadmium isotope fractionation in soil – cacao systems of Ecuador: a pilot field study

Fiorella Barraza[1,¥,*], Rebekah Moore[2,*], Mark Rehkämper[2,#], Eva Schreck[1], Grégoire Lefeuvre[1], Katharina Kreissig[2], Barry J. Coles[2], Laurence Maurice[1,3]

[1]Géosciences Environnement Toulouse (GET), Observatoire Midi-Pyrénées, Université de Toulouse, CNRS, IRD, 14 avenue E. Belin, F-31400 Toulouse, France.

[2]Department of Earth Science & Engineering, Imperial College London, London SW7 2AZ, UK.

[3]Universidad Andina Simón Bolívar, Área de Salud, P.O. Box 17-12-569, Quito, Ecuador.

* Joint first authors: Fiorella Barraza, fiorella.barraza@hotmail.com; Rebekah Moore, r.moore13@imperial.ac.uk
# Corresponding author: Mark Rehkämper, markrehk@imperial.ac.uk

[¥]Current affiliation: Instituto de Cultivos Tropicales (ICT), Tarapoto, Peru

Contents

1. Materials and Methods (detailed information).. 3

1.1 Study site locations.. 3

1.2 Collection and initial treatment of cacao and soil samples 3

1.3 Sample digestion 4

1.4 Validation of digestion methods.. 5

1.5 Cd isotope measurements 5

1.6 Error propagation 6

2. Soil properties 7

3. References 8

Figures and tables

Figure S1. Locations of Cacao Farms A, B and C in Ecuador.. 3

Table S1. Microwave program used for digestion of cacao samples 5

Table S2. Comparison of Cd concentrations measured by Q-ICP-MS for certified reference materials with reference values 5

Table S3. Cadmium concentration and isotope compositions obtained by MC-ICP-MS for reference and quality control materials and comparison with published results 6

Table S4. Detailed physicochemical data for the soil samples collected at the five sampling sites at three farms...7

1. Materials and Methods (detailed information)

1.1 Study site locations

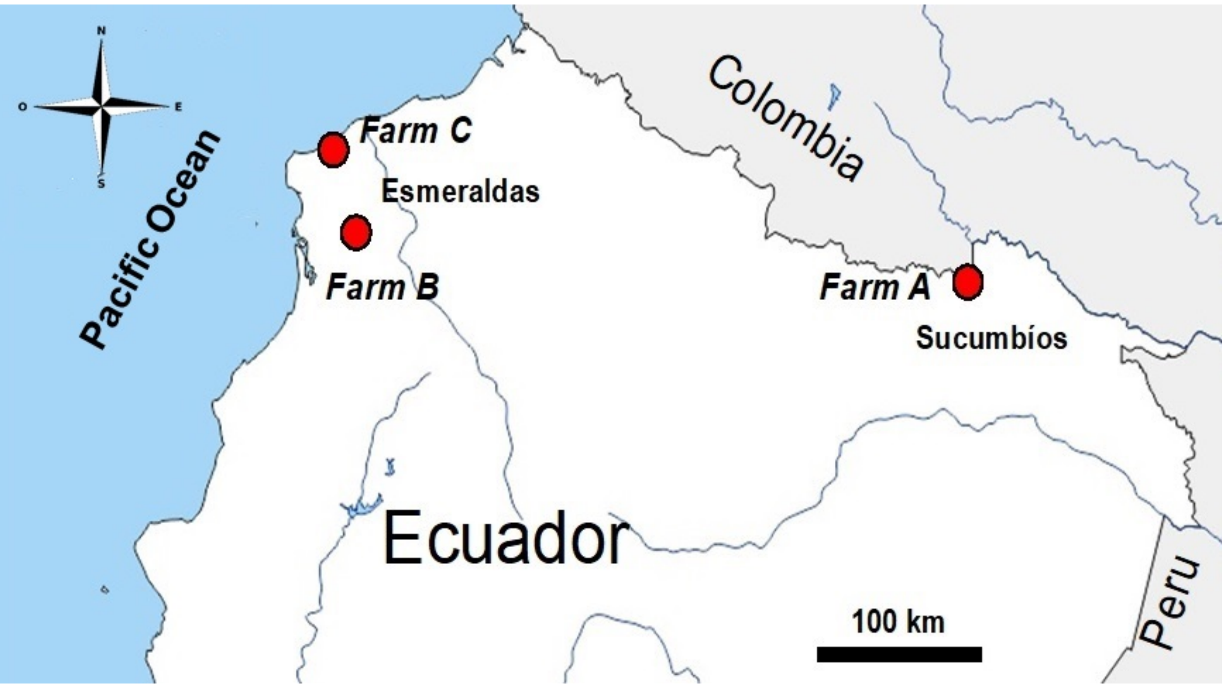

Figure S1. Locations of the cacao Farms A, B and C (see Table 1) in the Ecuadorian provinces of Sucumbíos and Esmeraldas where the soils, organic soil amendments and cacao samples were collected. Map source: https://d-maps.com.

1.2 Collection and initial treatment of cacao and soil samples

Similar sampling methods were employed at all sites, which followed established practices (Barraza et al., 2017). In detail, one mature and healthy cacao pod and ten mature leaves were collected randomly, either from the main trunk of the trees and/or from different branches. Samples were thereby taken from 3 different trees at each site, and these were mixed prior to further processing. In addition, between one and four soil profiles were collected at each location at a distance of 60 to 100 cm from the sampled trees using a stainless steel soil auger. The individual samples obtained for each depth interval (e.g., 0-20 cm) were combined to prepare a representative composite sample for each site for analysis.

The samples from Farm A were collected by the first author (F.B.) and freeze dried in a laboratory before shipping to France. The collected leaves were fresh, green and mature. The bean samples were not fermented in the field and were later ground with the shells for analysis.

All samples from Farms B and C were provided by the French cooperative ‘Ethiquable’ and they were originally collected for an independent study to investigate the effects of

agricultural practices and cacao processing on the Cd concentrations of beans. Following collection, these samples were shipped to the GET Laboratory in Toulouse for analysis. The leaves received from Farms B and C were a mixture of green and yellow mature leaves. The cacao beans were removed from the pods at the farms and then fermented and dried in the field without removal of the shells prior to shipping. Once received, the beans were further processed as received, without removal of the shells. The natural tree litter samples from Farms B and C were collected from underneath the sampled cacao trees and encompassed mainly leaves but also bark, twigs and pod husks, which were homogenized before analysis.

The two samples of cocoa liquor (also known as cocoa mass) from Sites A-1 and A-2 of Farm A were produced on the farm using the following procedure (see also Barraza et al., 2017):

- Cacao beans from each site were separated from pod husks and placed into mesh bags for transport to a stockpile for each site.
- At the stockpile, beans were fermented for 6 days (CCN-51 hybrid cacao) or 2 days (Nacional hybrid cacao) on wooden trays.
- The beans were then dried at ambient temperature for 3 days, cleaned and selected manually for toasting.
- Following toasting for two hours, the beans were again manually selected and milled for 2 hours.
- The bean coats (or shells) were discarded by processing in a peeling machine for 30 min.
- The cocoa liquor that was subsequently produced by heating was collected in plastic molds and chilled for 30 min.

At the GET Laboratory in Toulouse, all samples were first air and then oven-dried at 45° C. Subsequently, the samples were homogenized using either a vibrating cup mill with a steel grinding set and agate discs (soils, chicken manure, tree litter, cacao leaves, pod husks) or in an agate mortar with liquid nitrogen (cacao beans with shells, cocoa liquor).

1.3 Sample digestion

Soil and chicken manure samples were digested on a hotplate using 11 ml aqua regia and 3 ml HF in Savillex vials. A CEM Discover® SP high-pressure microwave system was employed to digest the cacao samples using a 3-stage heating protocol (Table S1) with 9 ml 15.6 M bi-distilled HNO_3, 1 ml H_2O_2 and 0.2 ml HF in quartz vials protected with Teflon PFA liners.

Table S1. Microwave program used for digestion of cacao samples

Stage	T (°C)	Time* (min)	P (bar)	Power (W)	Stirring
1	130	3	30	300	Med
2	160	1.5	30	300	Med
3	180	3	30	300	Med

*15 min in total, including cooling of samples

If any organic residue remained at this stage (as shown by a yellow to green colour of the solutions), the solutions were dried and the residues refluxed with 1 ml 15.6 M HNO_3 on a hotplate at 130° C until total digestion was achieved (as indicated by a transparent solution). Following evaporation and re-dissolution in 19.5 ml of 2% bi-distilled HNO_3, the sample solutions were aliquoted for the subsequent analyses.

1.4 Validation of digestion methods

A number of standard reference materials (SRMs) from NIST were analysed by Q-ICP-MS for quality control of the digestion and Cd recoveries are summarized in Table S2.

Table S2. Comparison of reference values and Cd concentrations measured by Q-ICP-MS for certified reference materials

Reference material	Description	n	Cd reference (mg kg^{-1})	Cd measured (mg kg^{-1})	Recovery (%)
NIST SRM2709a	San Joaquin Soil	4	0.356 ± 0.010	0.343±0.001	96
NIST SRM2384	Baking chocolate	3	0.0734±0.0077	0.0638±0.0004	87
NIST SRM1515	Apple leaves	3	0.013±0.002	0.012±0.002	92

n = number of individual samples that were digested and analyzed. The uncertainties denote 1sd of n analyses.

1.5 Cd isotope measurements

Following column chemistry, the purified Cd of each sample was dissolved in an appropriate volume of 0.1 M HNO_3 to obtain Cd solutions with a concentration of about 50 to 100 ng ml^{-1} for the Cd isotope measurements. At a sample uptake rate of ~120 µl min^{-1}, a sensitivity of about 200 V/(µg/ml^{-1}) or more was typically achieved for Cd using Faraday cups with 10^{-11} Ω resistors for data acquisition by MC-ICP-MS.

The raw measured ion beam and electronic baseline intensities from the runs were processed off-line with an iterative procedure to calculate the $\delta^{114/110}$Cd values of the samples, relative to bracketing runs of the NIST SRM 3108 Cd isotope reference material (Ripperger et al. 2007; Xue et al., 2012)

A sample of the BAM I012 Cd isotope reference material was fully processed through the sample preparation procedure with each batch of samples. Additional analyses were carried out for two environmental SRMs from NIST, Baking Chocolate SRM 2384 and San Joaquin Soil SRM 2709a, as well as an internal quality control material prepared from cacao leaves (Table S3).

Table S3. Cadmium concentrations and isotope compositions obtained by MC-ICP-MS for reference and quality control materials and comparison with published results.

Reference Material	Description	n	Cd ± sd (mg kg^{-1})	Cd Reference (mg kg^{-1})	$\delta^{114/110}$Cd ± 2sd (‰)	$\delta^{114/110}$Cd Ref. (‰)
BAM-I012[a]	Cd solution	4	--	--	-1.33 ± 0.07	-1.33 ± 0.04[b]
NIST SRM 2709a	Soil	2	0.353 ± 0.036	0.356 ± 0.010[c]	-0.17 ± 0.05	-0.22 ± 0.02[d]
NIST SRM 2384	Chocolate	2	0.0645 ± 0.0011	0.0734 ± 0.0077[c]	-0.26 ± 0.10	--
MAGIC NA-702[e]	Cacao leaves	1	102	106 ± 20	0.50 ± 0.06	0.56 ± 0.15

n = number of individual samples that were digested and analyzed. [a] Analyzed following processing through the column chemistry. [b] Mean result from Abouchami et al. (2013) [c] From certificate of the SRM. [d] Mean result from Wiggenhäuser et al. (2016). [e] In-house quality control material prepared from cacao leaves, whereby the plant was grown in a hydroponic solution containing added Cd. The (unpublished) reference values for this material represent the mean of nine results obtained on six digested sample aliquots that were previously analyzed in the MAGIC Labs; the quoted errors are 1sd and 2sd for the Cd concentration and isotope composition, respectively.

Notably, the analyses conducted during the course of this study produced results that are within error of the available reference values for the Cd isotope compositions and concentrations for all samples except the NIST SRM 2381 baking chocolate (Table S3). In the latter case, no isotope reference value is available whilst the Cd reference concentration is slightly higher than the Cd content determined here by isotope dilution and MC-ICP-MS. The latter result is, however, identical to the Cd concentration determined independently for the same sample solutions by Q-ICP-MS (Table S2).

1.6 Error propagation

The apparent isotope fractionation between two samples or reservoirs was calculated as:

$$\Delta^{114/110}\mathrm{Cd}_{\mathrm{A-B}} = \delta^{114/110}\mathrm{Cd}_{\mathrm{A}} - \delta^{114/110}\mathrm{Cd}_{\mathrm{B}}$$

where A and B denote the two Cd reservoirs. The individual 2sd measurement errors of the $\delta^{114/110}$Cd values (2sd-δCd) were propagated as follows to quantify the 2sd uncertainty of the calculated apparent isotopic fractionation:

$$2\mathrm{sd}\text{-}\Delta\mathrm{Cd}_{\mathrm{A-B}} = \sqrt{(2\mathrm{sd}\text{-}\delta\mathrm{Cd}_{\mathrm{A}})^2 + (2\mathrm{sd}\text{-}\delta\mathrm{Cd}_{\mathrm{B}})^2}$$

2. Soil properties

Table S4. Detailed physicochemical data for the soil samples collected at the five sampling sites.

**Depth (cm)**	**pH**	**CEC (cmol kg^{-1})**
Site A-1		
0-5	6.98±0.10	29.5±0.9
5-20	6.73±0.10	39.5±1.2
20-60	6.87±0.04	41.4±1.2
60-80	6.32±0.30	35.6±1.0
Site A-2		
0-5	6.28±0.09	31.2±1.0
5-20	6.65±0.04	42.6±1.3
20-60	6.96±0.10	38.0±1.3
60-80	6.57±0.35	41.7±1.3
Farm B		
0-20	6.30±0.09	7.7±0.2
Site C-a		
0-20	7.14±0.11	2.9±0.1
Site C-b		
0-20	7.08±0.10	1.7±0.1

The uncertainties denote 1sd of multiple analyses.

3. References

Abouchami, W., Galer, S.J.G., Horner, T.J., Rehkämper, M. Wombacher, F., Xue, Z. Lambelet, M., Gault-Ringold, M., Stirling, C.H., Schönbächler, M., et al. A common reference material for cadmium isotope studies - NIST SRM 3108. *Geostand. Geoanal. Res.* **2013**, *37*, 5–17. https://doi.org/10.1111/j.1751-908X.2012.00175.x.

Barraza, F.; Schreck, E.; Lévêque, T.; Uzu, G.; López, F.; Ruales, J.; Prunier, J.; Marquet, 515 A.; Maurice, L. Cadmium bioaccumulation and gastric bioaccessibility in cacao: a field 516 study in areas impacted by oil activities in Ecuador. *Environ. Pollut.* **2017**, *229*, 950–963. https://doi.org/10.1016/j.envpol.2017.07.080.

Ripperger, S., Rehkämper, M. Precise determination of cadmium isotope fractionation in seawater by double dpike MC-ICPMS. *Geochim. Cosmochim. Acta* **2007**, *71*, 631–642. https://doi.org/10.1016/j.gca.2006.10.005.

Wiggenhauser, M., Bigalke, M., Imseng, M., Müller, M., Keller, A., Murphy, K., Kreissig, K., Rehkämper, M., Wilcke, W., Frossard, E. Cadmium isotope fractionation in soil–wheat systems. *Environ. Sci. Technol.* **2016**, *50*, 9223–9231. https://doi.org/10.1021/acs.est.6b01568.

Xue, Z., Rehkämper, M., Schönbächler, M., Statham, P.J., Coles, B.J. A new methodology for precise cadmium isotope analyses of seawater. *Anal. Bioanal. Chem.* **2012**, *402*, 883–893. https://doi.org/10.1007/s00216-011-5487-0.
